# Close of the Volume

**Published:** 1860-08

**Authors:** 


### Close of the Volume.

With this number we close the fifth volume of the Atlanta Medical & Surgical Journal, and we would take the occasion to remind our subscribers that though the subscription for the Journal has been understood to be in advance, a large amount of indebtedness has accumulated upon our books, which we are not disposed to increase. It is, therefore, expected that those who desire the next volume of the Journal, will send on their subscriptions previous to the issue of the next number. From causes beyond control, our accounts have been thrown into some confusion, and if, therefore, any mistakes occur in rendering bills, (which is very probable), we hope to be excused, especially as the statement and correction of the *subscriber* will be regarded as satisfactory.

